# Paraoxonase 1 and atherosclerosis

**DOI:** 10.3389/fcvm.2023.1065967

**Published:** 2023-02-16

**Authors:** Paul N. Durrington, Bilal Bashir, Handrean Soran

**Affiliations:** ^1^Cardiovascular Research Group, Faculty of Biology, Medicine and Health, The University of Manchester, Manchester, United Kingdom; ^2^Department of Diabetes, Endocrinology and Metabolism, Peter Mount Building, Manchester University NHS Foundation Trust, Manchester, United Kingdom

**Keywords:** paraoxonase 1, paraoxonase 1 activity, cardiovascular disease, high density lipoprotein, lipid peroxidation, PON1 polymorphism

## Abstract

Paraoxonase 1 (PON1), residing almost exclusively on HDL, was discovered because of its hydrolytic activity towards organophosphates. Subsequently, it was also found to hydrolyse a wide range of substrates, including lactones and lipid hydroperoxides. PON1 is critical for the capacity of HDL to protect LDL and outer cell membranes against harmful oxidative modification, but this activity depends on its location within the hydrophobic lipid domains of HDL. It does not prevent conjugated diene formation, but directs lipid peroxidation products derived from these to become harmless carboxylic acids rather than aldehydes which might adduct to apolipoprotein B. Serum PON1 is inversely related to the incidence of new atherosclerotic cardiovascular disease (ASCVD) events, particularly in diabetes and established ASCVD. Its serum activity is frequently discordant with that of HDL cholesterol. PON1 activity is diminished in dyslipidaemia, diabetes, and inflammatory disease. Polymorphisms, most notably Q192R, can affect activity towards some substrates, but not towards phenyl acetate. Gene ablation or over-expression of human *PON1* in rodent models is associated with increased and decreased atherosclerosis susceptibility respectively. PON1 antioxidant activity is enhanced by apolipoprotein AI and lecithin:cholesterol acyl transferase and diminished by apolipoprotein AII, serum amyloid A, and myeloperoxidase. PON1 loses this activity when separated from its lipid environment. Information about its structure has been obtained from water soluble mutants created by directed evolution. Such recombinant PON1 may, however, lose the capacity to hydrolyse non-polar substrates. Whilst nutrition and pre-existing lipid modifying drugs can influence PON1 activity there is a cogent need for more specific PON1-raising medication to be developed.

## Introduction

It is 20 years since our last review of the role of paraoxonase in atherogenesis (1). In that time much has been learnt regarding the strength of the relationship of serum paraoxonase activity with atherosclerotic cardiovascular disease (ASCVD). Despite this, all too frequently the involvement of paraoxonase in atherogenesis is still regarded as controversial. However, whilst some aspects of the role of paraoxonase may be as yet poorly understood, a great deal has been clearly established. That will be the subject of this review.

## The development of the concept that PON1 is anti-atherosclerotic

Paraoxonase was identified by Aldridge in 1953 as an enzyme present in serum with the capacity to hydrolyse diethyl *para*-nitrophenyl phosphate (2). It was originally termed “A” esterase to distinguish it from “B” esterases, such as acetylcholinesterase and butyrylcholinesterase, which are inhibited by diethyl *para*-nitrophenyl phosphate, an organophosphate. Diethyl *para*-nitrophenyl phosphate, which is now more commonly referred to as paraoxon, is the potent neurotoxin produced by the metabolism of the organophosphate pesticide, parathion. The “A” enzymes hydrolysing paraoxon (EC.3.1.8.1, aryldialkylphosphatase) have thus come to be known as paraoxonases. Paraoxonase, circulating in blood and tissue fluid now designated as paraoxonase 1 (PON1), was found to constitute the first line of defence against a panoply of organophosphate toxins, including insecticides and military nerve gasses. Because of its importance in toxicology, it was intensively studied most notably by the research groups headed by Furlong (3) in Seattle and Draganov and La Du (4) in Ann Arbor. However, it was not until the mid-1980’s that Mackness, working in the toxicology department at the University of Reading, demonstrated that the location of paraoxonase was almost exclusively on HDL (5). Because of the known association of low HDL with ASCVD, it was but a short step to discovering that the serum activity was diminished in myocardial infarction (MI) survivors (6). Towards the end of that decade Mackness joined the lipoprotein group in Manchester and we began to study PON1 in diseases associated with accelerated atherosclerosis. We soon discovered that its activity was diminished both in diabetes (7, 8) and familial hypercholesterolaemia (8).

The prevailing dogma to explain the epidemiologically observed inverse relationship between HDL and ASCVD was that HDL was critical for reverse cholesterol transport. The evidence that HDL is rate-limiting for this process in typical human atherosclerosis was and remains scant (9). However, there were reports that HDL might protect LDL against potentially atherogenic modifications to its structure. As early as 1979 it had been found that the cytotoxicity of LDL to human vascular smooth muscle and endothelial cells in tissue culture could be abolished, if HDL was also present (10). Later it was found that:

(a) HDL decreased lipid peroxidation products measured as thiobarbituric acid reacting substances accumulating on LDL during Cu^2+^- or endothelial cell—induced oxidation (11).
(b) HDL diminished the increase in electrophoretic mobility of LDL following Cu^2+^-induced oxidation (12).
(c) HDL decreased the accumulation of malondiadehyde in LDL during oxidation induced by Fe^2+^ or by prolonged incubation (13). Fogelman’s group in Los Angeles then found that HDL prevented the minimally oxidised LDL-induced migration of human blood monocytes through a layer of cultured endothelial cells (14).

**FIGURE 1:**
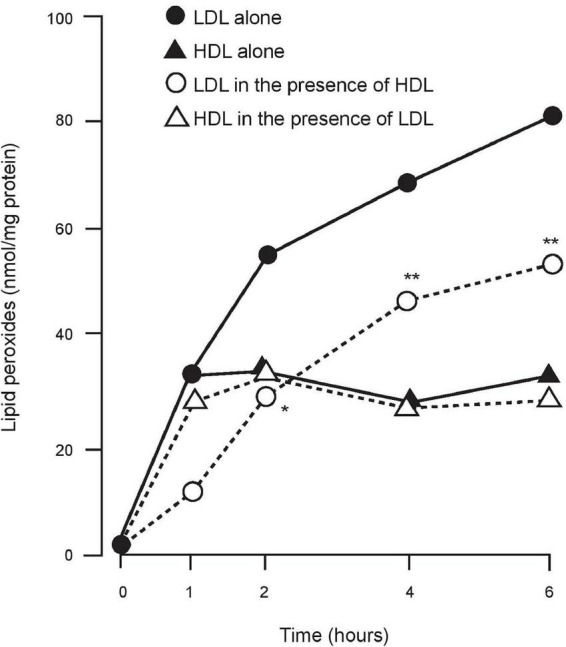
The accumulation of lipid peroxides on LDL and HDL when incubated alone and together in the presence of Cu^2+^. LDL + HDL significantly different from LDL alone. *P < 0.05; ^**^P < 0.001.

It was uncertain whether these effects (11–14) were due to transfer of lipid peroxides from LDL to HDL (probably for subsequent disposal by the liver) or was due to their breakdown by an enzyme present on HDL. Lecithin: cholesterol acyl transferase (LCAT) was considered for the latter role, but none of the groups was aware of the presence of PON1 on HDL. We began to speculate that PON1, which had no known physiological role, might be involved (15). We studied Cu^2+^-induced *in vitro* oxidation of LDL in the presence and absence of HDL, using the method of El-Saadani et al. to measure lipid peroxides (16) on LDL and HDL. We discovered in 1991 that Cu^2+^-induced LDL lipid peroxidation was not only less when co-incubated with HDL, but that it was also unaccompanied by any increase in lipid peroxides on the HDL and that the total lipid peroxides in the system were less when HDL was present (17; Figure 1). It was thus likely that HDL did not simply receive lipid peroxidation products from LDL, but that it also catalysed their conversion to products not detected as lipid peroxides in our assay. To explain this phenomenon, PON1 free of LCAT activity was isolated from HDL in lipid micelles and found to be a potent inhibitor of the accumulation of lipid peroxides on LDL when incubated with Cu^2+^ (17). A series of publications from our group followed supporting the hypothesis that PON1 was critical for the protection afforded to LDL against oxidative modification and extending this to include the concept that HDL, which is the predominant lipoprotein in tissue fluid, protected cell membranes against oxidative and other damaging processes (18–20). This seemed to provide an attractive anti-atherosclerotic role for PON1 on HDL. Interest in the oxidative theory of atherogenesis was, however, waning because of the failure of fat-soluble antioxidant vitamins to suppress atherosclerosis in clinical trials (see later) despite evidence that they prevent the formation of conjugated dienes in the initial stage of LDL lipid peroxidation by being more susceptible to oxidation than unsaturated fatty acyl groups. However, once they themselves are oxidised they are pro-oxidant and furthermore they increase cholesteryl ester transfer protein (CETP) activity (21, 22). PON1 on the other hand decreases the accumulation of the lipid peroxides generated after conjugate diene formation and does so over a longer time period.

In 1995 independent confirmation that prevention of oxidative modification of LDL by HDL was due to PON1 was provided by Fogelman’s group in Los Angeles (23). It was reported that the minimally oxidised LDL-induced migration of human blood monocytes through a layer of cultured endothelial cells was diminished in the presence of PON1 purified from HDL. Furthermore, by a mass spectrometry method, when purified PON1 was incubated with minimally oxidised LDL, it was found that oxidation products of phosphatidyl choline were decreased. Later, using electrospray ionisation mass spectrometry, we confirmed the marked decrease in histidine residues modified by 4-hydroxy-2-non-enal (HNE) (a break-down product of peroxidised linoleic acid typically present at the Sn2 position of phosphatidyl choline) in the tryptic fragments of LDL, which had been subject to Cu^2+^-induced oxidation when co-incubated with HDL possessing PON1 (24).

The oxidation products observed by the Los Angeles group to be present on minimally oxidised LDL in the absence of PON1 could themselves stimulate monocyte migration. The effect of PON1 in decreasing monocyte migration and phosphatidyl choline peroxidation products was enhanced by platelet activating factor acetyl hydrolase (PAFAH) (23). However, as we discuss later much of the PAFAH activity on HDL is probably due to PON1. Other factors proposed to explain the protective effect of HDL against lipid peroxide accumulation were LCAT and apo A1 (11, 25, 26). Experiments reported by our group showed that neither of these acting alone were effective in protecting LDL against lipid peroxidation in comparison to PON1 (27). However, enhancement of the effect of PON1 was observed from the addition of either LCAT or apo A1 to purified PON1 (27). This was greater after 4–8 h of co-incubation with LDL and PON1 than in the first 4 h (28). These observations were made under experimental conditions designed to determine whether PON1 has the capacity to prevent oxidative modification of LDL and, as we discuss later, the intensely hydrophobic environment created on HDL produced by the presence of apoA1 and the action of LCAT in converting pre-beta HDL to more mature HDL (29) is likely to be more critical *in vivo* for PON1 to exert its anti-oxidative, anti-atherogenic role. These and other HDL components which may contribute to anti-oxidant activity have recently been reviewed by us (30). However, in support of an important role for PON1, genetic deficiency of neither apoA1 nor LCAT in humans is, unlike PON1 deficiency, conspicuously associated with premature atherosclerosis (31, 32). Susceptibility to experimental atherosclerosis in *APOA1* or *LCAT* ablated mouse models requires an additional mutation, such as apoE deficiency or LDL receptor deficiency (33, 34) and even then may involve decreased PON1, whereas *PON1* knock-out mice are prone to atherosclerosis induced simply with atherogenic chow even without cross-breeding with apolipoprotein E (apoE) ablated mice (35).

Under oxidising conditions, regardless of the presence or absence of LDL, lipid peroxides begin to form on HDL at an early stage, but their accumulation then ceases remaining at a low level relative to LDL (Figure 1). In 1998 a consortium in Ann Arbor and Haifa (36) showed in experiments, involving enrichment of HDL with purified PON1 and specific inhibition of PON1 present in HDL, that the resistance of HDL to lipid peroxidation was due to its PON1 component.

To test our theory that HDL by virtue of its PON1 component might have a wider role by providing a system to protect cell membranes against oxidative damage, we determined the concentration of PON1 in experimental blister fluid as a surrogate for interstitial fluid (37). PON1 concentration was approximately one fifth that in serum and, although it was still associated with apoA1, the ratio between the two had decreased which was interpreted as likely to be due to sequestration of PON1 by the tissues. Later James’ group in Geneva showed that PON1 could exchange between HDL and outer cell membranes where it decreased cellular susceptibility to loss of function induced by oxidising conditions (38). This fitted well with our earlier immunohistochemical study of atheroma in the human aorta (39). ApoA1, clusterin (apoJ), and PON1 were found to be present in healthy coronary arteries, staining with increasing intensity as atheroma progressed (39).

It would be wrong to create the impression that the concept that PON1 can explain the anti-oxidative activity of HDL has not been without criticism. Firstly, a persisting effect of HDL to prevent LDL oxidation even when no PON1 activity can be detected, for example in the presence of EDTA (40) and secondly, failure of highly purified or recombinant PON1 to protect LDL against lipid peroxidation (41–43) have been interpreted as denying the theory. These assertions have been challenged by direct experimentation (44). Furthermore, the evidence that PON1 activity was absent due to inhibition by EDTA was based on measurements made using phenyl acetate as the substrate (40). Hydrolysis of phenyl acetate by PON1 is highly Ca^2+^-dependent whereas PON1 anti-oxidant activity can persist even in the presence of EDTA (45). Also, in experiments where highly purified or recombinant PON1 did not protect LDL against oxidative modification, it can be argued that in purifying PON1 to a high degree, whether from serum or tissue culture fluid, it is extremely difficult to maintain a lipid environment in which the conformation of PON1 necessary for its anti-oxidant activity can be maintained (41). Water-soluble PON1 mutants are even less likely to interact with the lipid environment physiologically to provide hydrolytic activity against highly hydrophobic substrates. Interestingly too, recombinant PON1 has cytotoxic properties (44) most likely due to its PAFAH-like activity in producing lysophosphatidyl choline (46), which, when it occurs outside the safe environment of HDL, is intensely damaging to tissues.

## PON gene family and PON1 polymorphisms

Whilst the major paraoxonase in serum is PON1, it was found that there are two other members of its family whose genes cluster on chromosome 7 (47). Paraoxonase 2 (PON2) is a widely distributed, highly expressed intracellular enzyme, which contributes to the intracellular anti-oxidant defences. Paraoxonase 3 (PON3) is another member of the paraoxonase family located on HDL, but at much lower concentration than PON1. It has very limited arylesterase and practically no organophosphatase activity. As lactonases, the substrate specificity of PON1 and PON3 overlap, but differ by degree with PON3 showing a preference for higher molecular mass lactones.

Paraoxonase 1 is highly polymorphic and it was found even before the advent of gene sequencing technology that at least one of these polymorphisms conferred variation in activity to different substrates (48). This variation in activity did not apply when phenyl acetate, which has a high molar rate of hydrolysis compared to other substrates including paraoxon. In the case of paraoxon, however, the frequency distribution of PON1 activity in Europid population revealed that almost half have a low activity, around 8–9% high activity and the rest form an intermediate peak. The heritability of these activities led to the discovery that PON1 was allelic with low activity and high activity homozygotes in Hardy-Weinberg equilibrium with heterozygotes. By the early 1990’s sequencing of PON1 and *PON1* revealed that this difference in activity was largely determined by a substitution of glycine (Q) for arginine (R) at position 192 resulting in a 192Q isoenzyme with low PON1 activity and a 192R isoenzyme with high activity. This gene variant is also known as rs662. It should be noted that the activity of these isoenzymes was reversed with some substrates other than paraoxon, such as diazoxon (49). Although naturally humans are unlikely to be exposed to paraoxon or diazoxon, these examples suggested the polymorphism may have evolved to permit a population to withstand a wider range of toxins than would otherwise be the case (see later). The prevalence of 192R and Q alloenzymes varies in different populations and tends to reflect their typical hydrolytic activity towards paraoxon (48).

In 1995 Ruiz and colleagues reported that in type 2 diabetes the 192R isoenzyme of PON1 was associated with coronary heart disease (50). Initially this seemed counter-intuitive, because the hydrolytic activity of this isoenzyme, at least with paraoxon as substrate, is higher than that of 192Q. The explanation proved to be that, per mg of HDL protein, 192R was slightly less effective than the 192Q alloenzyme in protecting LDL against lipid peroxidation (51, 52). In other words, the anti-atherosclerotic activity of PON1 is greater for the isoenzyme which is less effective in hydrolysing paraoxon. None the less the 192R isoenzyme does have anti-oxidative activity and, if present at high concentration, it will protect perhaps more than in an individual expressing the 192Q polymorphism at lower concentration. Although the polymorphism at position 192 does not affect PON1 serum concentration, others for example at position 55 (53) and in the promoter region (54) do and there are a huge number of epigenetic and acquired factors, including diabetes, inflammatory disorders, infections and nutrition, reviewed elsewhere (55, 56) that contribute to variation in concentration and activity which in different individuals can vary by as much as 16-fold and 40-fold, respectively (57).

## Epidemiology: Mendelian randomisation and serum PON1 activities

In epidemiological studies EDTA plasma is typically stored, but to test the association between PON1 activity and atherosclerosis when phenyl acetate, paraoxon, diazoxon, or a lactone are employed as substrate, serum is required, because all these activities are highly Ca^2+^-dependent (1). Genotyping, however, which can be done on any stored material likely to yield DNA, has since the 1990’s become increasingly easy. We were amongst the groups whose results were negative (58) with respect to an association between the Q allele and ASCVD risk, but others reported positively. By 2001 our meta-analysis showed a weak association between the Q allele and ASCVD, which was approximately what would be expected from our experiments (51, 52). In a subsequent meta-analysis by Wheeler et al., producing similar findings (59), it was considered that the association could also be explained by publication bias, with which we agreed (58). However, these authors concluded that their findings made it unlikely that PON1 was critical in atherogenesis. This was based on the unrealistic assumption that a gene coding for variation in PON1 activity towards paraoxon could provide results interpretable according to the principles of Mendelian randomisation. For this to be the case the influence of the 192 variants on atherosclerosis should have been similar in magnitude to its effect on *in vitro* paraoxon hydrolysis. The conclusion from meta-analysis of the 192 polymorphisms must be that it does not deny the hypothesis that low PON1 is associated with atherosclerosis: either the 192 genotype contributes nothing (publication bias) or it is supportive. Subsequently, meta-analyses, some without publication bias (60) have continued to show a small contribution of the 192Q allele to ASCVD risk, most obviously when diabetes is also present (60–62).

**FIGURE 2:**
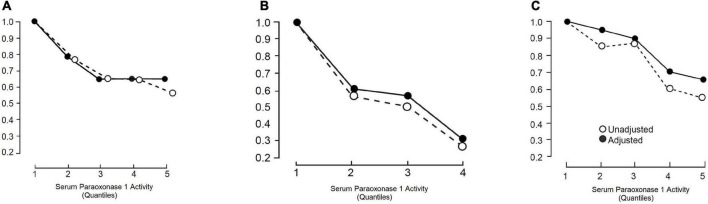
The risk of ASCVD relative to the lowest risk quartile or quintile (RR) as a function of serum paraoxonase 1 (PON1) activity studied prospectively in **(A)** Caerphilly and Speedwell (CHD only) (65), **(B)** cleveland clinic (ASCVD and all-cause mortality) (72), and **(C)** meta-analysis by Kunutsor et al. (ASCVD) (70). Closed circles are RR unadjusted for other risk factors and open circles after adjustment for some of these (see references for details).

**FIGURE 3:**
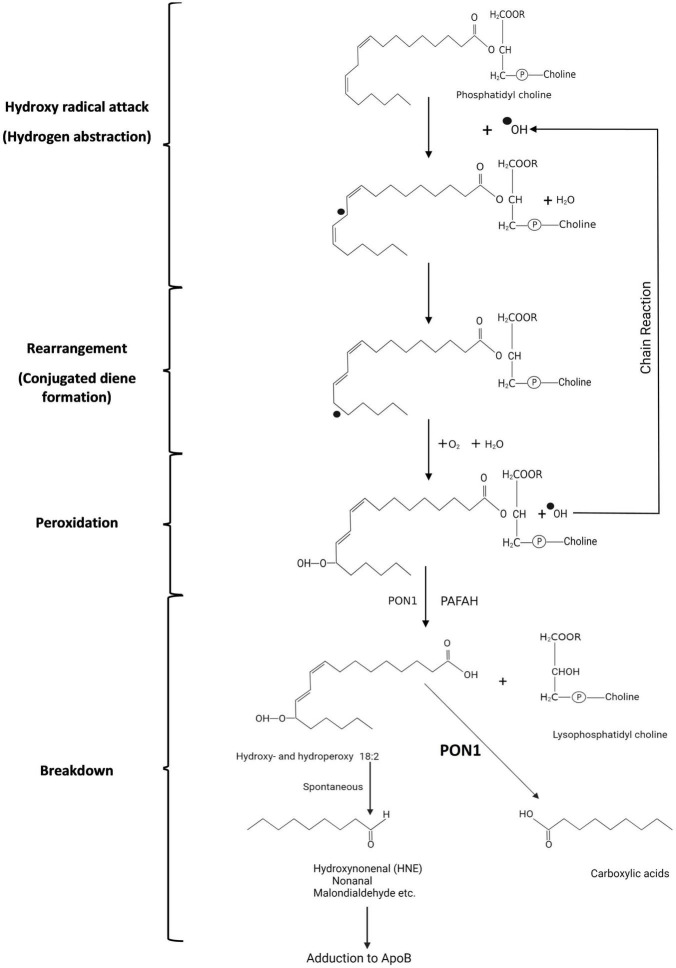
Symbolic representation of the peroxidation of phosphatidyl choline with linoleate in Sn2 by reactive oxygen species (hydroperoxy radical in this example). A superscript dot preceding an atom indicates an unpaired electron. (1) Hydrogen abstraction from the hydrocarbon chain leads to (2) rearrangement in which the double bonds between carbon 6 and 9 are no longer separated by 2 single bonds but by only one (conjugated diene formation). (3) Addition of oxygen then leads to peroxy radical formation with the potential for initiating a chain reaction and (4) spontaneous breakdown to aldehydes and ketones which can adduct to apolipoprotein B of LDL altering its receptor-binding. When PON1 in HDL is present breakdown to carboxylic acids rather than aldehydes and ketones is believed to occur.

After the initial case-control study showing an association between PON1 activity and myocardial infarction (6), we performed another study in which it was found that serum PON1 activity was already low in samples taken within 2 h of the onset of symptoms of acute myocardial infarction (63). There followed other case-control studies in which serum PON1 activity was, as expected, more closely related to the presence of ASCVD than PON1 genotypes (58, 64). The critical epidemiological test of whether PON1 activity was relevant to future atherosclerosis, however, came with reports of prospective studies. Serum PON1 was found to be independently associated with the likelihood of future ASCVD events, generally contributing to variation in risk with a similar magnitude to established risk factors, including HDL cholesterol (65–70). The first prospective results came from the Caerphilly Heart Study (65) of middle-aged men (Figure 2). Kunutsor et al. performed a meta-analysis (70) of these results combined with those of an additional five reports (65–69). There were 15,064 participants and 2,958 incidences of ASCVD. In three studies paraoxon was employed as substrate (65, 66, 69) and in three phenyl acetate (67, 68, 70). The age-adjusted pooled relative risk for ASCVD per 1SD higher PON1 activity was a 0.87, which was highly statistically significant. There was no evidence of publication bias. The literature in general does not reveal a close correlation between PON1 activity and HDL cholesterol or apoA1. Kunutsor et al. (70), however, did not find that PON1 activity could contribute more than HDL cholesterol to a multivariate equation to predict the likelihood of future ASCVD events. That could have been, because individual variation in serum PON1 activity is greater than for HDL for cholesterol and thus regression dilution bias would favour HDL cholesterol. The potentially greater biological significance of PON1 and discordance between HDL cholesterol and PON1 was emphasised by the report of Corsetti et al. of decreased PON1 activity in people with premature ASCVD despite high HDL cholesterol levels (71). In the meta-analysis by Kunutsor et al. (70) PON1 measured as aryl esterase activity (phenyl acetate hydrolysis), which is unaffected by the 192 polymorphism, predicted ASCVD more strongly than paraoxonase (paraoxon hydrolysis) activity. PON1 activity predicted new ASCVD events particularly strongly in people with established ASCVD, such as those who had undergone coronary revascularisation. The study by Bhattacharyya et al. (72) was of particular interest in the latter context. They studied 1,399 people who underwent coronary angiography at the Cleveland Clinic. PON1 measured both as aryl esterase and paraoxonase activity was strongly inversely associated with the incidence of new ASCVD event over a minimum follow-up of 3 years (Figure 3). In a subgroup of 150 participants matched for the PON1 192 polymorphism (equal numbers with the QQ, QR, and RR) serum PON1 activity was strongly correlated (*P* < 0.001) with concentrations of fatty acid oxidation products (hydroxyeicosatetraenoic acid, HETE; hydroxyloctadecadienoic acid, HODE, and 8-isoprostane prostaglandin F2α, 8-isoPGF_2α_).

Because of the theory that PON1 has evolved as a lactonase (see later), it has been proposed that use of a lactone substrate, such as homocysteine thiolactone or dihydroxycoumarin (73, 74) might provide a more biologically relevant estimate of PON1 activity. There is as yet no prospective epidemiological evidence that the lactonase activity of PON1 provides a superior indicator of ASCVD risk (75). The significance of PON1 lactonase activity in human disease is emergent territory (76). Specifically, in the case of atherosclerosis some evidence suggests homocysteine thiolactone, which, like glucose and lipid peroxides, can bind to apoB, may be associated with ASCVD incidence (77). Urinary homocysteine thiolactone excretion has been reported to be increased in people with low serum PON1, particularly in carriers of the PON1-192R allele (73).

## Evolution and biological plausibility

Polymorphisms of PON1, by broadening the range of organophosphate neurotoxin resistance, will increase the survival of an exposed population without the need to await a new mutation with greater detoxifying properties, albeit at the expense of individuals with the less favourable variant (78). Darwinian evolution takes vastly longer and extinction may occur before a more successful mutation occurs. In this context, we have reported that agricultural workers involved in sheep-dipping with diazinon (active metabolite diazinoxon) are less likely to experience neuropsychiatric symptoms if they possess the 192Q PON1 allele associated with higher serum PON1 measured as diazoxonase activity (79). The organophosphates which led to the discovery of PON1 were synthetic, but a vast array of organophosphates is naturally produced. The habitat of the earliest hominids was on the shores of the great lakes of Africa, where cyanobacteria (blue-green algae) (80), which can produce large quantities of neurotoxic organophosphates, at times, would have threatened human survival. However, modern man has been present for a mere 6 million years, not long enough to explain the evolution of the paraoxonase family of proteins, the ancestral protein for which may have existed hundreds of millions or even billions of years ago. It is likely that PON1 evolved from PON2, its intracellular relative (49). Beyond that we know that paraoxonases are not homologous to serine esterases, carboxyesterases, or arylesterases and thus do not have similar ancestry (49). The capacity to synthesise cholinesterases and to respond to acetyl choline dates to before metazoa emerged and the evolution of any recognisable nervous system (81). The potential for organophosphate toxicity must have been present for at least as long. Organophosphates produced in the anaerobic conditions around deep sea hydrothermal vents must have been incorporated into the earliest life forms. So it may not be too fanciful to consider that the ancestral protein giving rise to paraoxonases may have existed many aeons ago and have long had a role in organophosphate metabolism. Other examples of enzymes with organophosphatase activity conserved across the domains of living organisms are: diisopropylfluorophosphatase (DFPase) (eukaryocyte squid) (82), organophosphate hydrolase, organophosphate acid anhydrolase and phosphotriesterase (bacteria) (83) and SsoPox an organophosphatase/lactonase from *Sulfolobus solfataricus* (archea) (84). Of these the structure of rePON1 resembles that of squid DFPase. Both are six-bladed propellers with each blade consisting of four β-sheets. Moreover, in both structures two calcium ions can be found in their central tunnel (82).

With the advent of photosynthetic organisms an atmosphere rich in oxygen was created and thus the scene was set for the evolution of life with more rapid metabolism (energised by oxidative respiratory chain phosphorylation) than could be sustained by simple glycolysis. However, simultaneously the necessity for protection against the toxicity of oxygen also became essential. Paraoxonases and the other anti-oxidative enzymes would have contributed to that (85).

Elias and Tawfik in a fascinating review have strongly argued that paraoxonases may have evolved, not as esterases, but as lactonases with a promiscuous esterase activity (86). Although not related in other aspects of their structure, their active site has features more in common with lactonases than esterases. Many single-celled organisms signal to each other by producing lactones, such as *N*-acylhomoserine lactones, usually when their colony size has reached some critical point (quorum sensing), altering expression of genes regulating such processes as bioluminescence, biofilm formation, virulence factor expression, and motility. Just as for a hormone to excite rather than inhibit there must be a process to destroy it after receptor binding (ironically, for example, acetyl choline and acetyl cholinesterase), so a lactonase could have a role in quorum sensing. PON1 has the capacity to metabolise homocysteine thiolactone, which has been implicated in atherogenesis (87). However, this activity is dwarfed beside that of biphenyl hydrolase-like protein, making a critical role for ancestral PON1 in that regard less likely (73, 76, 77, 88).

The view that PON1 may have a more generalised role in the immune system has been proposed by Camps et al. (89) based on their finding of an increase in chemokine (C-C motif) ligand 2 (CCL2) production in PON1 deficiency. CCL2 induces migration and infiltration of immune cells into target tissues in a range of inflammatory disorders, which could include the arterial wall.

An apparently quite different role for PON1 which might have selective advantage is its capacity to inactivate gram negative bacterial endotoxin (90). This endotoxin is a lipopolysaccharide, which introduces yet another class of substrates which PON1can hydrolyse with important biological consequences.

Thus paraoxonases appear to have diverged from other enzymes early in evolution. They display great substrate promiscuity and their primary function (organophosphatase, anti-oxidant, lactonase, lipopolysaccharidase) may have been different at various times in evolutionary history and in different classes or even orders of living organisms. Myocardial infarction was unknown before the 20th century (91). It is thus inconceivable that PON1 has evolved to combat atherosclerosis. Nonetheless it is very possible that an enzyme which has provided survival success in some other context might by virtue of its promiscuity protect against ASCVD (“wide substrate specificity” might be better terminology than “promiscuity” when considering virtue).

## Evidence from animal experiments that PON1 protects against atherosclerosis

Birds do not express serum PON1 and they are not only susceptible to organophosphate poisoning, but their HDL lacks the capacity to impede the accumulation of lipid peroxides in human LDL under oxidising conditions (92). Mammals display serum PON1 activity, although with considerable species variation (93). Experimental atherosclerosis in rodents has provided consistent evidence that PON1 infusion or over-expression can suppress atherogenesis or that inhibition or ablation of the PON1 gene promotes atherogenesis. Thus the *PON1* knockout mouse, which, like birds is susceptible to organophosphate toxicity, also produces HDL which has a diminished capacity to protect LDL against oxidative modification. It is susceptible both to atherosclerosis induced nutritionally and by apoE deficiency (35). Consistent with this, rabbits fed an atherogenic diet developed more advanced atherosclerotic lesions when PON1 activity was inhibited with nandrolone (94). As long ago as 2002 a US patent was registered (95) for the prevention of atherosclerosis by injection of a preparation of PON1 192Q isoform based on a mouse model. Over-expression of PON1 has been achieved in mouse (96–100), rabbit (101–103), and rat (104) models. Such experiments have consistently revealed decreased susceptibility to atherosclerosis and enhanced HDL functionality. Ablation or over-expression of the PON1 gene causes little if any effect on lipoprotein metabolism, unlike knock out or overexpression of major genes sometimes considered to be important in atherogenesis, such as apoA1 and LCAT. The small decrease in blood pressure in PON1 deficiency (105) would be expected to oppose rather than increase atherogenesis. Thus, a major role for PON1 in atherogenesis is the only plausible explanation for the results of animal experiments. Furthermore, in experiments to test the contribution of risk factors other than PON1 to atherosclerosis, the atherogenic diet used in, for example rabbits, would have decreased PON1 and contributed to atherogenesis from whatever other cause was being examined (106).

## Physical and structural properties of PON1

For its antioxidative activity towards lipid hydroperoxides, PON1 requires an intensely hydrophobic environment. Its molecular structure contains long sequences of hydrophobic amino acids creating regions eschewing water and allowing PON1 to exist within the lipid-rich domains of HDL. The strong detergent properties of apoA1 (107) are likely to be crucial in this respect. The hydrophobicity of PON1 makes it resistant to crystallisation. Removed from its lipid environment, naturally occurring PON1 is unstable and tends to aggregate in the absence of detergents. This is true whether wild-type PON1 (wtPON1) is isolated from serum or from the culture medium of *E coli* expressing wtPON1. This has had two major effects on progress in research into the involvement of PON1 in atherosclerosis. Firstly, its structure remained the subject of speculation, which was unresolved until it was submitted to directed evolution in order to increase its solubility (108). Secondly, as methods were developed for increasing purification of wtPON1, dispute ensued about how much of its capacity to prevent the accumulation of lipid peroxides on LDL was retained (41–44).

Family shuffling of four PON1 genes (human, mouse, rabbit, and rat) resulted in many variants that could be expressed in *E. coli*. One of them produced crystals of a quality suitable for X-ray diffraction studies (108). This was the recombinant-PON1 (rePON1) G2E6 variant, which exhibits 91% homology to the wt rabbit PON1 and 86% homology to the human PON1 (109). Structural analysis using X-ray crystallography revealed the six-bladed β-propeller structure of PON1 with a central tunnel that houses two calcium ions. There is a unique addition to the β-propeller scaffold in the form of three α-helices, which are located on the top of the propeller. These helices are likely to be involved in anchoring of PON1 to HDL particles.

Each calcium ion, depending on its location within the enzyme, plays an important part in the activity of PON1. The calcium ion located closer to the tunnel entrance has a structural role that may be necessary for some conformational aspects of PON1 important for some of its substrates, such as organophosphates. It may be less critical to, say phospholipids, with which it is surrounded within HDL. The other calcium ion which lies deep in the active site cavity has a catalytic role and is important for substrate positioning and ester bond activation. Differences in the active site configuration and positioning of a calcium ion are likely to be important in explaining the differential substrate specificity of the 192 polymorphisms, but with such a wide spectrum of substrates no single mechanism may exist (110). Engineered variants with increased aqueous solubility and tagged to simplify purification have at the time of writing led to 21 rePON1 products being available from 8 suppliers. Generally, the evidence that these retain enzymic activity in initial screening has been based on phenyl acetate hydrolysis. The commercial incentive has been to produce rePON1 variants for the treatment of organophosphate poisoning or prevention. The aim has thus been to create rePON1’s that are more active in hydrolysing organophosphate neurotoxins than wtPON1, which is less active than squid DFPase, a rival target for bio-engineering. The hydrophobicity of rePON1 must necessarily have been diminished. This may not impair its capacity to hydrolyse molecules such as phenyl acetate and simple organophosphates, but hydrolysis of more intensely hydrophobic long chain fatty acyl lipid substrates may be abolished.

## Mechanism by which HDL and PON1 act to protect against lipid peroxide accumulation

Polyunsaturated fatty acyl groups are susceptible to peroxidation due to oxygen free radicals leaking from cells or deliberately produced in the tissue fluid by inflammatory cells (myeloperoxidase, NADPH oxidase). In the human, linoleate (C18:2) is the major circulating polyunsaturated fatty acid, typically occurring at the Sn2 position of phosphatidyl choline. The earliest phase of lipid peroxidation is hydrogen abstraction. This leads to conjugated diene formation detectable by ultraviolet spectroscopy (111). The double C = C bonds (outer orbitals occupied by an electron from a single hydrogen) in linoleate are separated by two single C-C bonds (outer orbitals occupied by electros from two hydrogens). A conjugated diene is formed when one of the double bonds flips over, because one of its hydrogen atoms is attracted by hydroxyl radicals (OH^●^ created by the reaction of O_2_^●^ with water) with their unpaired electrons (Figure 3). The newly created double bond is separated from the next double bond by a single bond (conjugated diene), an assemblage which resonates with ultraviolet light. This early phase of lipid peroxidation is opposed by chain-breaking lipid soluble antioxidants, such as α-tocopherol (vitamin E), β-carotene, and ubiquinol-10, which offer themselves as electron donors in preference to linoleate, but it is largely unaffected by PON1. The major effect of PON1 present in HDL is in decreasing the accumulation of lipid peroxides in LDL derived from these conjugated diene free radicals in the later phase of the oxidation of linoleate (21). The failure of clinical trials with, for example, vitamin E (112) is frequently seen as dismissing the oxidative modification of LDL theory of atherosclerosis. However, it is not at the early stage of conjugated diene formation that LDL is chemically modified, allowing arterial wall macrophage and smooth muscle cell receptor-mediated uptake (111). This occurs later when oxygen becomes bound to linoleate causing its decomposition firstly into, for example, 9-hydroperoxy-10,12-octodecadienoic acid (9-HPODE), and thence aldehydes (e.g., propanedial, hexanal, nonanal), unsaturated aldehydes (e.g., hexenal, nonenal) and their various hydroperoxy derivatives. It is these aldehydes which adduct to the side chains of proline and arginine residues of apoB, leading to fragmentation of the apoB molecule, which thereby becomes a ligand for macrophage and transformed smooth muscle cell scavenger receptors, such as scavenger receptor class B type 1 (SCARB1) (111, 113–119). Possibly some derivatives of oxidised polyunsaturated fatty acids have a steric resemblance to lactones (120).

For PON1 to protect LDL or cell membranes against aldehyde adduction it must come into physical contact with their phosphatidyl choline and cholesteryl ester components. In tissue fluid this may be achieved through the engagement of HDL particle with outer cell membranes (38, 121). In the circulation, particularly in humans in whom cholesterol esterification occurs on HDL due to the action of LCAT, huge amounts of phospholipid and of free and esterified cholesterol are transferred between HDL and apoB-containing lipoproteins. This process is greatly facilitated by cholesteryl ester transfer protein (CETP) and phospholipid transfer protein (PLTP) (122–124). Esterification of cholesterol by LCAT yields one molecule of the highly cytotoxic lysophosphatidyl choline for every molecule of cholesterol esterified (125). HDL retains this lysophosphatidyl choline safely until it passes through the hepatic sinusoids where it is released to hepatocytes for re-esterification.

**FIGURE 4:**
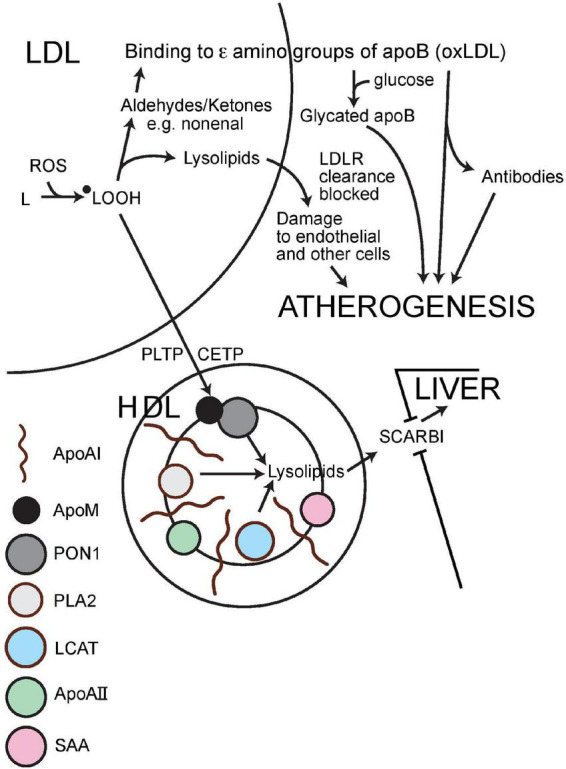
Schematic representation of the mechanism by which HDL impedes the atherogenic modification of LDL. ApoA1, apolipoprotein AI; ApoAII, apolipoprotein AII; apoB, apolipoprotein B; ApoM, apolipoprotein M; PON1, paraoxonase 1; PLA2, phospholipase A2 (syn: PAFAH, platelet activating factor hydrolase); LCAT, lecithin cholesterol acyl transferase; SAA, serum amyloid A; PLTP, phospholipid transfer protein; CETP, cholesteryl ester transfer protein; SCARB1, scavenger receptor class B1; oxLDL, oxidatively modified LDL; LDLR, LDL receptor; L, lipid; ^●^LOOH, hydroxylipid radical; ROS, reactive oxygen species (containing oxygen with an unpaired electron giving it an outer shell resembling fluorine).

Arriving on HDL, phosphatidyl choline with, as the consequence of oxygen free radical attack, fatty acyl hydroperoxide/conjugated dienes at Sn2, the initial phase of detoxification is likely to be the release of these fatty acyl molecules from Sn2 by the PAFAH (platelet activating factor; syn phospholipase A2, PLA2)-like activity of PON1. Quite possibly this is facilitated not at the lactonase active site deep in the catalytic tunnel of PON1, but perhaps more superficially and might even take place to some extent spontaneously. It is, however, likely that the oxidised linoleate products released can reach the deeper PON1 catalytic site (whatever its teleology) (45) where they are converted to harmless carboxylic acids as opposed to reactive aldehydes (20, 23, 126; Figure 4). This concept of PON1 activity has been challenged by a report that highly purified PON1 lacks both PAFAH activity and the capacity to prevent the accumulation of phosphatidylcholine oxidation products (41). Nonetheless, as has been previously discussed, PON1 divorced from HDL may not be able to hydrolyse intensely hydrophobic substrates and there is no denying the antioxidant activity of intact HDL or the evidence from gene manipulation that PON1 makes a crucial contribution to this.

## Epigenetic factors and modulators of PON1 activity

A host of diseases and nutritional factors are associated with variation in PON1 activity. These and potential mechanisms for their associations with PON1 have recently been reviewed (55, 56). Both dyslipidaemia (8, 127–134) and diabetes mellitus (8, 135–145) are associated with decreased activity (see later). The composition of HDL has a major effect on PON1 activity. In inflammation HDL has decreased PON1 activity (146–148). At the same time the apolipoprotein AI and clusterin (syn. apolipoprotein J) content of HDL also diminish whereas apoliporotein AII (apoAII) and serum amyloid A (SAA) increase (146–149). The resulting pro-inflammatory HDL has decreased antioxidant capacity. SAA increases in inflammation. Experimentally SAA can displace PON1 from HDL (148) but the mechanism for the replacement of PON1 by SAA in pro-inflammatory HDL may also involve inhibition of hepatic PON1 expression and stimulation of that of SAA (146–150). The antioxidant activity of PON1 is also limited by myeloperoxidase (MPO), a pro-oxidant enzyme secreted by neutrophils (151–155) to kill bacteria by showering them with oxygen free radicals. MPO is taken up by HDL where it may form a complex with PON1. The anti-oxidative activity of HDL may thus reflect a balance between those two. Bearing in mind that HDL is the dominant lipoprotein in tissues, it may not be fanciful to speculate that it may operate to confine pro-oxidant activity to sites of inflammation and to limit the spread of oxygen free radicals systemically. When infection or inflammation becomes generalised, as in, say, septicaemia (90, 156) or systemic lupus erythematosus (157) PON1 activity has declined.

## Diabetes and metabolic syndrome

Both type 1 and type 2 diabetes are associated with decreased PON1 activity and low PON1 activity is related to both macro- and microvascular complications (8, 135–145). Furthermore low PON1 may predispose to the development of type 2 diabetes (143, 158, 159).

It is reported that *in vitro* glycation of PON1 by incubation with glucose at high concentration decreases its activity (160, 161). This, of course, cannot explain the low serum PON1 activity in metabolic syndrome (prediabetes) before the onset of hyperglycaemia. What is of much greater interest is the occurrence of glycated apoB in the circulation. In non-diabetic people some 4% of serum apo B is glycated and in diabetes the percentage is typically twice this (162). The concentration of glycated apoB is also raised when LDL is increased even in the absence of diabetes, for example in familial hypercholesterolaemia. The concentration of glycated apoB, whether in diabetic or non-diabetic people, is higher than oxidatively modified apoB (162, 163). ApoB in the smaller, denser subfractions of LDL (SD-LDL) is more heavily glycated than in VLDL and less dense LDL (164–166). This may be because more of the apoB molecule is exposed to glucose in SD-LDL or because it has a longer residence time in the circulation or both. Glycation of apoB occurs at the arginine and proline residues to which ketones and other derivatives of lipid peroxidation adduct (167). Glycated apoB, like oxidatively modified apoB, is taken up by macrophages to form foam cells (168). *In vitro* apoB in LDL is resistant to glycation. Prolonged incubation of normal LDL with high concentrations of glucose does raise its level, but not usually to the same extent as is found in diabetes (165, 168). The explanation may be that the more highly oxidative environment *in vivo* allows prior adduction of, say aldehydes to arginine, which is then replaced by glucose (166, 169) or a more reactive derivative of glucose, such as gluconolactone or methyl glyoxal, is generated during glycolysis (170–172). Either mechanism might suggest a possible effect of HDL in protecting LDL apoB against glycation which has been reported *in vitro* with HDL from people with above median serum PON1 activity opposing glycation more than HDL from those with lower activity (173). More work is needed to explore the possibility that HDL might protect against atheroma and more specifically diabetic complications by this mechanism.

## Future directions: Therapeutic and diagnostic potential

There is a plethora of nutritional studies of PON1 activity. Unsurprisingly, given the different substrates used to measure PON1 activity, the small size and the inadequate design of many, findings often appear conflicting or unconvincing. No amount of meta-analysis can provide clarification. The impression gained is that obesity is often associated with decreased PON1 activity, albeit most obviously when triglycerides are raised or diabetes is present (127, 128, 132–145, 174). It is also clear that HDL cholesterol concentration is often discordant with changes in PON1 activity. The Mediterranean diet may increase PON1 activity (175) and various fruit juices, most conspicuously pomegranate juice, can raise PON1 activity (176). Dyslipidaemia, whether due to familial hypercholesterolaemia or hypertriglyceridaemia, is associated with diminished PON1 activity (8, 127, 128, 130–134) with perhaps its most profound decreases occurring in familial dysbetalipoproteinaemia [unpublished observation]. Statin (177), fibrate (178), ezetimibe (179), probucol (128), niacin (180), and metformin (181) drugs raise serum PON1 activity whilst sulphonamides may decrease it (182).

A pharmacological approach to raising PON1 activity is attractive, but traditionally it is easier to block rather than activate enzymes. Raising HDL by CETP inhibition was ineffective in preventing atherosclerosis except by its LDL lowering effect (183). CETP may be necessary for the transfer of oxidised phospholipid and cholesteryl ester to HDL for PON1 to act on them and the HDL particles created are large (184) and not the smaller, desirable particles rich in PON1 capable of facilitating cholesterol efflux. PON1-rich HDL infusion is probably not a practical possibility, particularly as rePON1, which is easily produced may have little anti-oxidant capacity [see earlier]. Evidence suggests that HDL mimetics, some of which could be given orally, can raise PON1 activity in particles resembling physiological HDL (185, 186). It will also be important to be aware of effects on PON1 of the various antisense oligonucleotides for lowering LDL and triglycerides as they emerge. There also exists the theoretical possibility of raising PON1 activity by promotion of its gene or expression of a gain-of-function variant (but without a polar tag so that it is incorporated physiologically into HDL) (187).

Paraoxonase 1 has the potential to contribute to the clinical assessment of ASCVD risk. However, continuing uncertainties about identification of the substrate critical in its anti-atherosclerotic activity have slowed progress in that direction. Is it important, for example, to employ a long chain fatty acid peroxide or lactone rather than, say, phenyl acetate or paraoxon as the substrate in an assay? However, whilst discovery of the key substrate(s) in the mechanism by which PON1 protects against atherosclerosis is essential for our understanding of its role, this may not be critical to make use of it clinically. Alkaline phosphatase is one of the most frequently requested and informative biochemical tests in clinical practice, but its physiological role remains obscure, and the substrate used in its measurement artificial (188). Currently, measurement of PON1 hydrolytic activity has generally been more closely associated with ASCVD than PON1 protein concentration, because the specific activity of PON1 is variable, for example in diabetes (see earlier discussion). PON1 hydrolyses phenyl acetate at a much higher rate than paraoxon. The PON1 192 polymorphism does not affect the hydrolysis of phenyl acetate but does that of paraoxon. On the other hand, if the true physiological role of PON1 is its lactonase activity, then potentially there could be advantages to using lactones, such as dihydrocoumarin or homocysteine thiolactone, as assay substrates (73–75). However, this has yet to be proven. Undoubtedly too, mistakes, such as the use of plasma rather than serum and inclusion of B esterase and non-specific hydrolysis in some methods for determination of A esterase (PON1) activity, have led to some confusion. A carefully conducted laboratory investigation using a variety of candidate substrates (189–192) linked to an epidemiological study is required.

Measurement of serum PON1 activity also provides an indication of where the HDL present in individuals is in the spectrum of pro- to anti-inflammatory and pro- to anti-atherosclerotic capacity (100, 193). Cholesterol efflux capacity is another indicator, but its measurement is more difficult and more prone to error (194). Because decreased PON1 activity is frequently associated with increased SAA in HDL the ratio of SAA concentration to PON1 activity has been proposed as better index of the type of HDL present than either measurement singly (148).

## Conclusion

Serum PON1 activity is inversely associated with ASCVD incidence both in human and animal studies. Although this was discovered due to the presence on HDL of PON1 and its contribution to the anti-oxidative capacity of HDL, the lactonase activity of PON1 may also contribute to the mechanism by which it reduces ASCVD risk. PON1 provides a potential additional means of clinical ASCVD risk assessment and is an indicator of the extent to which HDL has retained its anti-atherogenic and anti-inflammatory properties. It has the potential for therapeutic exploitation.

## Author contributions

PD conceptualised the study, performed the literature search, wrote the draft, and finalised the manuscript. BB and HS performed literature searches, contributed to writing, revised the draft, proofread the manuscript, and designed the figures. All authors contributed to the article and approved the submitted version.
